# Ameliorative Effects of Phytochemical Ingestion on Viral Infection in Honey Bees

**DOI:** 10.3390/insects11100698

**Published:** 2020-10-13

**Authors:** Edward M. Hsieh, May R. Berenbaum, Adam G. Dolezal

**Affiliations:** Department of Entomology, University of Illinois at Urbana-Champaign, 505 S. Goodwin, Urbana, IL 61801, USA; maybe@illinois.edu (M.R.B.); adolezal@illinois.edu (A.G.D.)

**Keywords:** honey bee, *Apis mellifera*, phytochemical, caffeine, Israeli acute paralysis virus, survival

## Abstract

**Simple Summary:**

Virus infection is among the many stressors honey bees are experiencing in the modern agricultural landscape. Although some promising treatments are currently under development, no reliable cure currently exists. Here, we investigated the effects of various phytochemicals (plant-produced chemical compounds) on the survivorship of virus infected honey bees. Our results showed that, when consumed at natural concentrations like those found in flowers, caffeine is capable of significantly reducing the mortality of infected bees. It is important to note that caffeine did not clear the infected bees of all viruses and should, therefore, not be considered a virus cure. Rather, caffeine represents a potential antiviral therapeutic agent that should be studied further to improve understanding of virus-phytochemical interactions.

**Abstract:**

Honey bee viruses are capable of causing a wide variety of devastating effects, but effective treatments have yet to be discovered. Phytochemicals represent a broad range of substances that honey bees frequently encounter and consume, many of which have been shown to improve honey bee health. However, their effect on bee viruses is largely unknown. Here, we tested the therapeutic effectiveness of carvacrol, thymol, *p*-coumaric acid, quercetin, and caffeine on viral infection by measuring their ability to improve survivorship in honey bees inoculated with Israeli acute paralysis virus (IAPV) using high-throughput cage bioassays. Among these candidates, caffeine was the only phytochemical capable of significantly improving survivorship, with initial screening showing that naturally occurring concentrations of caffeine (25 ppm) were sufficient to produce an ameliorative effect on IAPV infection. Consequently, we measured the scope of caffeine effectiveness in bees inoculated and uninoculated with IAPV by performing the same type of high-throughput bioassay across a wider range of caffeine concentrations. Our results indicate that caffeine may provide benefits that scale with concentration, though the exact mechanism by which caffeine ingestion improves survivorship remains uncertain. Caffeine therefore has the potential to act as an accessible and inexpensive method of treating viral infections, while also serving as a tool to further understanding of honey bee–virus interactions at a physiological and molecular level.

## 1. Introduction

Honey bees are a keystone species in the modern crop pollination landscape, generating an estimated global annual value of over $215 billion USD in agricultural production [1,2], but recent years have seen increasing challenges to the beekeeping industry [3,4]. Honey bees suffer from a combination of biotic and abiotic stressors, including pesticides, poor forage, and diseases, particularly viruses transmitted by the ectoparasitic Varroa mite (*Varroa destructor*) [5,6,7], all of which can lead to high rates of hive loss and subsequent replacement [8].

Although developing technologies used to combat viruses have been successful to some extent [9,10] or show great promise [11], to date, there are currently no available treatments or medicines to treat honey bee virus infection and management focuses mainly on reducing Varroa loads.

In addition to developing pharmaceutical approaches to treat viruses, there is also evidence that natural components of the honey bee diet can provide protection against pathogens. For example, the diversity and quality of pollens consumed by individual bees can have a significant effect on their ability to tolerate infections [12,13,14]. Individual secondary plant metabolites, or phytochemicals, are also routinely collected by foragers in nectar, pollen, and resins, and are typically present in honey [15]. Many of these phytochemicals have immune-boosting effects in *Apis* species and may even serve as part of a self-medication strategy among social bees [14].

In recent years, there has been rising interest in the use of essential oils as both nutraceuticals, i.e., food components that impart physiological benefits, typically to humans, and antimicrobial control agents in other animals [16,17]. Thyme oil, derived from *Thymus vulgaris*, and oregano oil, from *Origanum vulgare*, both confer immune-boosting benefits in humans [17,18,19] through their primary active ingredients, the isomeric monoterpenes thymol and carvacrol, respectively. Thymol, which inhibits growth of a wide variety of bacterial and fungal species and has been used for a broad range of traditional medicinal purposes [17], is also the active ingredient in the popular Varroa control treatments Apiguard^®^ and ApiLife VAR^®^ [20,21]. Moreover, feeding on thymol reduces deformed wing virus (DWV) levels in young bees, increases antimicrobial peptide expression in older bees [22], and suppresses the development of the trypanosome parasite *Crithidia bombi* [23].

The phenolic acid *p*-coumaric acid (PCA) and the flavonol quercetin both belong to the group of phytochemicals that are ubiquitous in pollen, propolis, and honey [24]. Both, when ingested, upregulate genes that encode cytochrome P450 monooxygenase enzymes, which are responsible for the detoxification of xenobiotics [25,26,27]. PCA also upregulates antimicrobial peptide gene expression when fed to larval and adult bees [25,28] and improves honey bee survivorship when infected with *Nosema ceranae* microsporidian gut parasites [29]. Both PCA and quercetin have also been shown to possess antiviral qualities; however, these studies have primarily been conducted in human pathogen models (e.g., cell culture systems with model human viruses) [30,31] and not pollinators that normally collect and consume these phytochemicals. For example, PCA inhibited the replication of human rhinovirus [31], which belongs to the order *Picornavirales*, to which many of the more frequently encountered bee viruses (DWV, Israeli acute paralysis virus or IAPV, Black queen cell virus or BQCV, and others) also belong [32]. These commonalities, in conjunction with the anti-DWV effect observed by Palmer-Young et al. [22], suggest that there may be a similar antiviral result when PCA and quercetin are administered to honey bees.

Perhaps one of the most widely recognized plant secondary metabolites, caffeine, is an alkaloid naturally found in the seeds, leaves, and berries of a wide variety of plant species, including coffee (*Coffea* spp.), cocoa (*Theobroma cacao*), and tea (*Camellia sinensis*) [33,34]. Apart from acting as an important stimulant for many humans, caffeine is capable of triggering a wide variety of behavioral and physiological reactions across a variety of taxa and is toxic to many herbivorous insects [33]. In spite of its toxic effects, caffeine can also be found in the floral nectar and pollen of *Coffea* and *Citrus* species [34,35], where its defensive qualities do not appear to deter pollinator feeding. In fact, both honey bees and bumble bees prefer sucrose solutions containing caffeine [36,37], and caffeine consumption improves learning and memory formation [35,38,39]. In the field, bees stimulated by a caffeine reward intensify their recruitment behaviors, increasing foraging and waggle dance frequency, as well as persistence to target dance location [40]. Despite the large body of research on behavioral effects of caffeine on honey bees, fewer studies have examined the physiological consequences of caffeine ingestion. Balieira et al. [41] showed that caffeine helps to ameliorate the oxidative stress induced by the neonicotinoid insecticide imidacloprid. Both Strachecka et al. [42] and Bernklau et al. [29] found that caffeine increased the lifespan of honey bees infected by *N. ceranae* by reducing spore loads. However, very few studies have examined the effects of caffeine on virus infection, with only one other study showing that it can inhibit DWV replication and upregulate immunity genes [43].

IAPV is particularly useful as an infection source in honey bee viral response assays. This virus has been linked to widespread colony losses [44] and can be transmitted through *Varroa* and oral routes. Although it is not quite as ubiquitous as more commonly studied viruses such as DWV, it can produce clearly discernable phenotypes in bees infected as adults, including lethargic movement, reduced reaction to stimulus, and eventual death [45]. Additionally, its effects on survivorship in controlled conditions is relatively well-characterized [9,13,46,47], meaning IAPV can serve as a valuable model virus for testing interactions with other factors, as it allows for high-throughput bioassays with standardized and repeatable laboratory protocols [48].

Here, we combined IAPV infection bioassays with exposure to five candidate phytochemicals (thymol, carvacrol, PCA, quercetin, caffeine). Due to their reported effects in other systems, and in some cases their potential for playing a role in plant-pollinator coevolution, we hypothesized that consumption of these phytochemicals in the diet would confer some level of protection to bees that experience a viral challenge. We predicted that feeding of the phytochemicals, in conjunction with a model infection assay with IAPV, would result in improved survival compared to controls.

## 2. Materials and Methods

### 2.1. Virus Particle Production

Large quantities of virus particles were produced and amplified inside honey bee pupae using methods similar to those compiled by de Miranda et al. [49] and Carrillo-Tripp et al. [47] with minor modifications [48]. While virus particles can be derived from adults or pupae, pupal propagation is very efficient at producing large quantities of concentrated virus [48,49]. Pupae were collected using two different methods: (1) pupal excision and (2) larval self-removal.

For pupal excision, brood frames containing white-eye pupae were selected and removed from healthy hives in the University of Illinois at Urbana-Champaign (UIUC) apiary (Champaign County, IL). White-eye pupae were gently excised using forceps and arranged in groups of 8–10 in Petri dishes lined with filter paper. All excised pupae were then injected with 1 µL of a 1% virus particle solution suspended in phosphate-buffered saline (PBS). The stock virus particles were identical to those described in Geffre et al. [50]. Injections were performed between the third and fourth abdominal tergites using a Combitip injector and a 30G needle. The Petri dishes were then stacked in a Tupperware container and placed in an incubator maintained at 34 °C and 75% relative humidity (RH) for approximately 3 to 5 days. Daily inspections were performed and dead or rotting pupae were removed before bacterial or fungal buildup could occur. After allowing sufficient time for the virus particles to propagate, the pupae were placed into 50 mL conical tubes, homogenized by vortexing, and stored in the −80 °C freezer until ready for extraction and concentration.

Brood extractions were completed with a second method: larval self-removal. This approach results in much higher throughput with no measurable difference in viral production. Instead of selecting brood frames containing pupae of an approximate age, honey bee queens were caged on an empty frame and allowed to lay eggs for 24 h. Following the egg-laying period, queens were released, and the egg frames were marked and allowed to develop normally within the colony. At exactly 192 h after caging (the point right before fifth instar larvae are capped), marked frames were transferred to an incubator maintained at 34 °C and 75% RH and laid face-down (larval cells facing downwards) onto plastic food containers lined with Kimwipes^®^ (Kimberly-Clark Professional, Roswell, GA, USA). Overnight, the food-seeking instinct of late-stage larvae drives them to crawl out of their cells and drop onto the padded containers below. The larvae were then removed from the containers and individually arranged onto shallow, plastic trays lined with Kimwipes and tented with aluminum foil to retain moisture. The trays were then returned to the incubator and larvae were allowed to develop for an additional 5 to 6 days until they reached the white-eye stage, whereupon they were injected and sampled following the steps outlined in the pupal excision protocol.

### 2.2. Virus Particle Extraction and Concentration

Virus particles were grown and extracted from eight separate colonies across 16 total samplings. The homogenized pupae gathered during viral production were thawed, transferred to centrifuge bottles, and mixed with approximately three volumes of 1× PBS on a shaker at room temperature for 10 min. The bottles were then centrifuged using a Sorvall RC-5B (Marshall Scientific, Hampton, NH, USA) ultracentrifuge equipped with a GSA Rotor (Marshall Scientific, Hampton, NH, USA) at 15,000× *g* for 5 min at 4 °C, after which the supernatant was decanted and filtered through cheesecloth to remove remaining fat globules. The supernatant was extracted using 0.3 volumes of 24:1 chloroform:isoamyl alcohol and centrifuged at 21,000× *g* for 20 min at 4 °C. The subsequent aqueous phase was then decanted into a beaker and RNAse-free water was added to bring the total volume to 200 mL. Beakers were then transferred to a 4 °C cold room and placed onto magnetic stir plates. 4.6 g NaCl and 14 g polyethylene glycol 8000 (PEG) were slowly added to each beaker under constant gentle stirring. The mixtures were stirred continually for an additional 5 h in the cold room, after which they were incubated overnight to allow virus particles to precipitate. Following incubation, the mixtures were transferred to clean bottles and centrifuged at 15,000× *g* for 30 min at 4 °C to recover a PEG-particle pellet. The pellet was then resuspended in TES buffer (10 mM Tris-HCl pH 7.5, 2 mM EDTA, 150 mM NaCL) and passed through an 18G needle ten times before being aliquoted into 2-mL centrifuge tubes. The tubes were centrifuged at 13,000× *g* for 15 min at 4 °C, after which the supernatant was separated and centrifuged again at the same settings to ensure total removal of all PEG. The remaining supernatant was then concentrated to approximately 2 mL using Amicon^®^ Ultra-4 Centrifugal Filter Unit (MilliporeSigma, Burlington, MA, USA) via centrifugation at 14,000× *g* for 10 min at room temperature. The concentrated particles were passed through a 26G needle and centrifuged one final time at 14,000× *g* for 5 min at room temperature. The viscous supernatant was then separated and stored at −80 °C until ready for use.

### 2.3. Virus Particle Purification and Quantification

RNA was extracted from the concentrated virus particles using TRIzol (Life Technologies, Carlsbad, CA, USA) extraction and DNA was removed using DNAse I (RNase-free) (New England BioLabs, Ipswich, MA, USA). Based on the protocols described by Carrillo-Tripp et al. [47], fragments of the IAPV genome were amplified using one-step reverse transcription (RT)-qPCR with the Power SYBR^®^ Green RNA-to-CT™ 1-Step Kit (Applied Biosystems, Foster City, CA, USA) following an absolute quantification approach using 100 ng total RNA per sample. Amplification was performed using a 384-well Quantstudio 6 Flex Real-Time PCR System (Applied Biosystems, Foster City, CA, USA) and programmed as follows: reverse transcription [48 °C-30 min], enzyme activation [95 °C-10 min], PCR–40 cycles of [95 °C-15 s, 60 °C-1 min], melt curve [95 °C-15 s, 60 °C-1 min]–[stepwise increases of 0.05 °C/s from 60–95 °C, hold for 15 min]. The final copy numbers were extrapolated using a 1:10 serially diluted RNA-based standard curve containing sequences of the viruses of interest [47].

### 2.4. Cage Assays: General Protocol

The following protocol describes the universal processes shared by all subsequent cage assays. Specific details regarding experimental treatments, treatment concentrations, sample size, and testing duration can be found in their corresponding subsections.

Five or more frames of bees on the verge of eclosion (<24 h) sourced from at least three separate hives located within the UIUC apiary were placed into emergence boxes and stored inside a 34 °C incubator set to 50% RH. All source colonies were closely monitored for mites throughout the experimental season. Any colony found to exceed a 2% mite infestation threshold in adult bees was excluded from the source pool until their mite loads returned to an acceptable range. After 24 h, all frames were removed and newly emerged bees were brushed into a single collection tub. In order to minimize the hive genetic effects of any individual colony, all bees were gently mixed to produce a homogeneous mixture before being separated into acrylic cages with 35 bees per cage. The cages were then transferred to the same incubator and randomly arranged to prevent any microclimate effects. Immediately following the transfer, each cage received a small weigh boat containing either 600 µL of 30% sucrose solution mixed with an IAPV inoculum or an equivalent quantity of sterile but otherwise untreated sucrose solutions (see associated subsections for concentrations of viral inocula employed in specific experiments). The IAPV inoculum used in all subsequent experiments involving a virus inoculation stage was generated according to the protocols by de Miranda et al. [49], Carrillo-Tripp et al. [47], and Hsieh et al. [48] and contained 9.86 × 10^7^ viral copies per 100 ng RNA composed of 99.79% IAPV with only trace levels of DWV, BQCV, and sacbrood virus (SBV). After allowing for total consumption of inocula within all cages (approximately 12–14 h), feeder tubes (15 mL Falcon tubes with 18G needle holes poked in the bottom) were inserted into each cage, the contents of which were provided *ad libitum* and refilled as necessary throughout the course of each experiment (see subsections for experiment-specific feeder solutions).

Mortality within every cage was recorded at 12 h intervals for the first 72 h of each experiment, following which recordings were switched to 24 h intervals. Dead bees were removed following each instance of mortality observation to avoid repeated count errors. Although bees were not sampled from every experiment, every experiment in which bees were sampled followed an identical protocol: three bees were haphazardly selected at 36-h post-inoculation (hpi) and placed into centrifuge tubes on dry ice. Dead bees were given priority over live bees for sampling to minimize depopulating the cages. The 36-hpi timepoint was selected as past experimentation has shown that this tends to be the point at which IAPV titers are highest within the bee without inducing lethal effects [13].

IAPV dose-establishment experiments were conducted in both field seasons (summers of 2018 and 2019) prior to phytochemical experimentation to select appropriate inocula concentrations. All experiments in 2018 (Experiments 1–3) used a 1% virus solution and all experiments in 2019 (Experiments 4–7) used a 0.01% virus solution.

### 2.5. Phytochemical Screening

Monoterpenes such as carvacrol and thymol are naturally repellent to honey bees [51,52] and were thus encapsulated within ß-cyclodextrin, a cyclic oligosaccharide, before being fed to IAPV-inoculated honey bees. All carvacrol solutions used in this and subsequent experiments were produced by mixing 30% sucrose solution with encapsulated carvacrol particles provided by Dr. Juan Andrade and his laboratory using the methods detailed by Gaur [19]. An experimental carvacrol dose of 160 ppm was selected following a dose-response experiment scaled around a previously tested concentration of thymol [22].

#### 2.5.1. Carvacrol Moderate Dose Treatment (Experiment 1)

We tested whether a 160 ppm carvacrol solution affects IAPV infection using the standard cage assay protocol. The experiment consisted of four treatments (1% IAPV inoculated and uninoculated controls, 1% IAPV inoculated and uninoculated 160 ppm carvacrol), ran for 7 days (168-hpi), with 7 cages per treatment, and no sampling was performed.

#### 2.5.2. Carvacrol Acute Rescue (Experiment 2)

Following the standard cage assay protocol, virally inoculated bees were fed elevated levels of carvacrol to determine whether the essential oil could act as therapeutic agent for cases of acute IAPV infection. The experiment consisted of an uninoculated control, two carvacrol concentrations (1000 and 2000 ppm) that were either inoculated or uninoculated with 1% IAPV, run for 3 days (72-hpi), *n* = 7 cages per treatment, and no sampling was performed.

#### 2.5.3. Thymol Acute Rescue, Prophylactic, and Regular Treatment (Experiment 3)

The effects of thymol supplementation were tested across three treatment forms: acute rescue response, *ad libitum* feeding, and prophylactic treatment. Both acute rescue (1000 ppm thymol) and *ad libitum* (160 ppm thymol) feeding treatments with or without 1% IAPV inoculation followed the standard cage assay protocol, running for 11 days (264-hpi), with 7 cages per treatment, and no sampling being performed (Table 1). The prophylactic treatments were tested using the same parameters but differed in their virus inoculation schedules. Instead of being fed a virus inoculum immediately after being separated into cages, bees in the prophylactic treatments received 160 ppm thymol-sucrose solution *ad libitum* (with controls instead receiving 30% sucrose) for 6 days before being fed the standard virus inoculum. Following inoculation, both treatments were switched to a sucrose-only diet and monitoring was performed as normal. All thymol particles and solutions were produced using the same methods as those of the carvacrol solutions.

### 2.6. Caffeine Investigation

#### 2.6.1. Differentiating Caffeine in Multiple Phytochemical Trial (Experiment 4)

Following the standard cage assay protocol, virus inoculated bees were subjected to three different phytochemical treatments with or without 0.01% IAPV inoculation: *p*-coumaric acid (82 ppm), quercetin (75.6 ppm), and caffeine (25 ppm). All feeder solutions contained 0.25% DMSO in order to solubilize *p*-coumaric acid (PCA) and quercetin [26]. Concentrations for PCA and quercetin were selected based on the natural range observed in local honey and beebread [26] and 25 ppm caffeine was selected based on the natural range in which the substance is found in *Citrus* and *Coffea* nectar [34,35]. The experiment included a sucrose control and ran for five days (120-hpi), with 10 cages per treatment and standard sampling. All treatments were evenly divided into two concurrent trials initiated within 24 h of one another for practical reasons.

#### 2.6.2. Caffeine Trial (Experiment 5)

To focus on the effects of caffeine on viral infection, a subcomponent of Experiment 4 was replicated by repeating only the control and caffeine treatments. The sample sizes were increased to 20 cages per treatment (80 cages total), but all other experimental parameters were held constant. Treatments were split into two concurrent trials (see Experiment 4).

#### 2.6.3. Caffeine Dose–Responses (Experiments 6A and 6B)

To determine honey bee reactions to a range of caffeine concentrations, two separate caffeine dose–response curves were generated: one testing IAPV inoculated bees and the other testing uninoculated bees. The inoculated dose–response trial (Experiment 6A) followed the standard cage assay protocol and tested four caffeine concentrations (25, 100, 1000, and 10,000 ppm) and a control (30% sucrose), all of which were inoculated with 0.01% IAPV. The test ran for 7 days (168-hpi), with 20 cages per treatment, and standard sampling was performed. The uninoculated dose–response trial (Experiment 6B) followed an identical protocol to Experiment 6A with the following two exceptions: (1) all cages received a virus-free inoculum containing only sucrose solution and (2) testing ran for 12 days (288-hpi). Caffeine is sufficiently soluble in sucrose solution so DMSO was not used in any of the treatments, as in Experiments 4 and 5. Both Experiments 6A and 6B were split into two concurrent trials (see Experiment 4).

### 2.7. Sampled Bee Homogenization for Viral Quantification

To confirm infection and address the question of viral tolerance vs. resistance, we quantified the viral titers of the caffeine-fed bees and their control counterparts sampled from Experiments 4 and 5. We purposefully selected only dead bees from all treatments for quantification to guarantee the selection of a known IAPV phenotype (i.e., death). In order to homogenize the sampled bees across all collected treatments, 8 cages were randomly selected with the use of a random number generator from each of the four treatments (caffeine +, − and control +, −) pooled across Experiments 4 and 5 for a total of 32 cages. From each cage, 2 out of the 3 sampled bees were selected for quantification; *n* = 64 samples. Dead bees were ‘simulated’ in the treatments with fewer than necessary dead-upon-collection samples (i.e., the uninoculated cages) by haphazardly selecting bees sourced from uninoculated treatments previously killed via freezing at −80 °C, placing them into empty cages, and transferring them to an incubator (34 °C, 50% RH). The selection of dead and simulated-dead bees minimizes the risk of introducing survivor bias by collecting live bees (i.e., sampling bees that were not subjected to increased levels of infection) and previous work has shown that not only are honey bee viruses capable of remaining infective outside of a honey bee for well over 12 h [53], dead bees can provide comparable patterns of infection to live bees [47]. Previous work has also shown that this method can successfully detect and estimate virus titers in dead individuals from many bee species [54], and comparison of live and dead inoculated bees revealed no significant differences in virus titers [47]. After 12 h of incubation, the simulated dead bees were removed from their cages and immediately processed for viral quantification using the previously stated methodology.

### 2.8. Statistical Analyses

Survival analyses in Experiments 1–3 were conducted using Cox proportional-hazards modeling performed in R using the survival (version 3.1-11; [55]) and survminer (version 0.4.6; [56]) packages. Experiments 4–6 were subdivided into two concurrent trials each that were initiated within 24 h of one other. Consequently, these experiments were analyzed using mixed effects Cox models in the coxme package (version 2.2-16; [57]) to account for the trial factor. Subsequent pairwise comparisons within all Cox models were corrected using Benjamini-Hochberg corrections to reduce Type I error rate. Viral loads obtained from RT-qPCR were estimated using QuantStudio RealTime PCR Software (Applied Biosystems, version 1.3, Foster City, CA USA, https://www.thermofisher.com/us/en/home/global/forms/life-science/quantstudio-6-7-flex-software.html) and then analyzed with R using the dplyr package (version 0.8.3; [58]). Normality assumptions were not met despite Box-Cox transformations, and thus the data were analyzed using the Kruskal-Wallis rank sum test. Pairwise differences were determined using Wilcoxon rank sum and multiple comparisons were corrected using Benjamini-Hochberg corrections. All graphics were generated in R using the ggsurvplot function within the survminer package (version 0.4.6).

## 3. Results

### 3.1. Phytochemical Screening

#### 3.1.1. Monoterpene Screening (Experiments 1–3)

**Figure 1:**
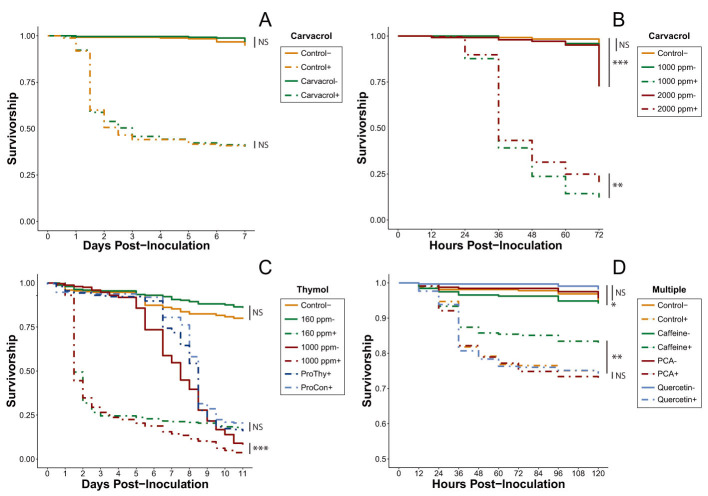
Mean survival curves of all honey bee cages inoculated (+, dash lines) or uninoculated (−, solid lines) with Israeli acute paralysis virus (IAPV) and fed different diets during the phytochemical screening process. Tested phytochemicals are noted above the legend. (**A**) Bees were fed sucrose solution or 160 ppm carvacrol-sucrose solution, *n* = 7 cages per treatment. (**B**) Bees were fed sucrose solution or high concentration carvacrol-sucrose solutions, *n* = 7 cages per treatment. (**C**) Bees were fed sucrose solution or varying concentrations of thymol-sucrose solution. “Pro” denotes prophylactic diet treatments (ProCon + = unamended sucrose; ProThy + = 160 ppm thymol solution) in which inoculation did not occur until day 5, *n* = 7 cages per treatment. (**D**) Bees were fed sucrose solution, 25 ppm caffeine, 82 ppm *p*-coumaric acid (PCA), or 75.6 ppm quercetin, *n* = 10 cages per treatment. NS, not significant; * *p* < 0.05, ** *p* < 0.01, *** *p* < 0.001 (pairwise Cox proportional-hazards models, Benjamini-Hochberg correction).

Overall, none of the tested carvacrol treatments improved the survivorship of IAPV- inoculated bees relative to their control treatments. Neither the inoculated (Cox proportional-hazards models; HR = 1.01, *p* = 0.964) nor uninoculated (Cox proportional-hazards models; HR = 0.61, *p* = 0.319) 160-ppm carvacrol treatments significantly altered survival compared to controls (Figure 1A, Table S1). The higher concentrations used in the acute rescue trial (1000 ppm, 2000 ppm) not only failed to improve survivorship, they increased the mortality rate (Figure 1B, Table S2). Surprisingly, the bees in the inoculated 1000-ppm treatment experienced significantly lower survival than those in the inoculated 2000-ppm treatment (Cox proportional-hazards models; HR = 0.90, *p* < 0.001), but the survivorship of the bees in the uninoculated 1000-ppm treatment was not significantly different from that of the control bees (Cox proportional-hazards models; HR = 1.68, *p* = 0.22) while the survivorship of bees in the uninoculated 2000-ppm treatment was significantly lower (Cox proportional-hazards models; HR = 2.03, *p* < 0.001).

None of the three thymol dosing strategies improved survivorship of IAPV-inoculated bees. Although there was no significant difference detected between the uninoculated 160-ppm thymol treatment and the control, hazard modeling showed a very strong trend toward improved relative survivorship in the thymol-fed cages (Cox proportional-hazards models; HR = 0.66, *p* = 0.065). By contrast, the mortality in the inoculated 160-ppm thymol treatment was much higher when compared to the carvacrol treatment at the same dose (Figure 1A,C, Table S3). Furthermore, the inoculated 1000 ppm thymol experienced significantly lower survivorship than the uninoculated 1000 ppm (Cox proportional-hazards models; HR = 2.69, *p* < 0.001), with bees in both treatments experiencing sharp declines in survivorship (~5% survival at Day 11), although this drop did not begin in the uninoculated group until Day 5 (Figure 1C). Additionally, thymol does not appear to provide any prophylactic protective effect. After viral inoculation on Day 5, survival rates of bees in both prophylactic treatments rapidly dropped and the trial concluded with no significant difference between the two treatments (Cox proportional-hazards models; HR = 1.16, *p* = 0.168).

#### 3.1.2. Multi-Phytochemical Screening (Experiment 4)

All of the treatments involving uninoculated bees in the multi-phytochemical trial were not significantly different from one another, with the exception of uninoculated bees consuming quercetin, which survived significantly better than uninoculated bees consuming caffeine (Cox proportional-hazards models; HR = 0.46, *p* < 0.001; Figure 1D). However, in the inoculated group, only the mortality risk for caffeine-fed bees differed significantly from the mortality risk in all other treatments, with their mortality risk reduced by 21.9% when compared to the inoculated control (Cox proportional-hazards models; HR = 0.78, *p* = 0.008; Table S4). As a result, caffeine was selected as the only phytochemical eligible for continued investigation.

### 3.2. Caffeine Investigation

#### 3.2.1. Caffeine Trials (Experiment 5)

**Figure 2:**
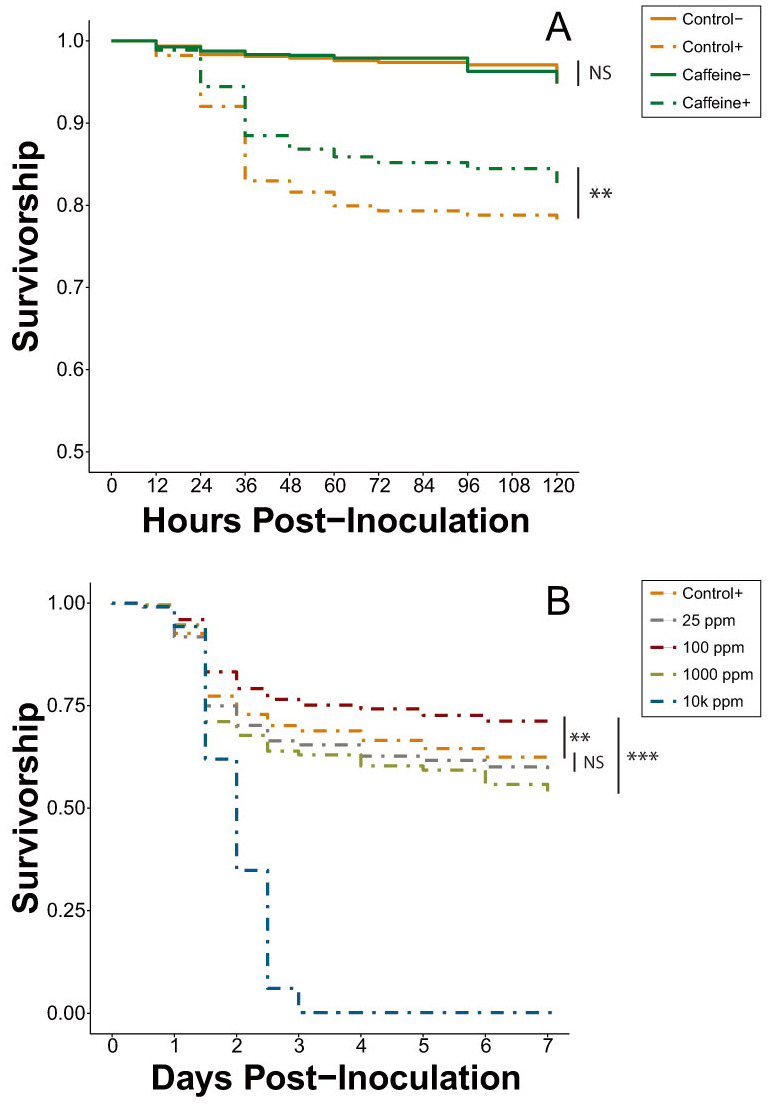

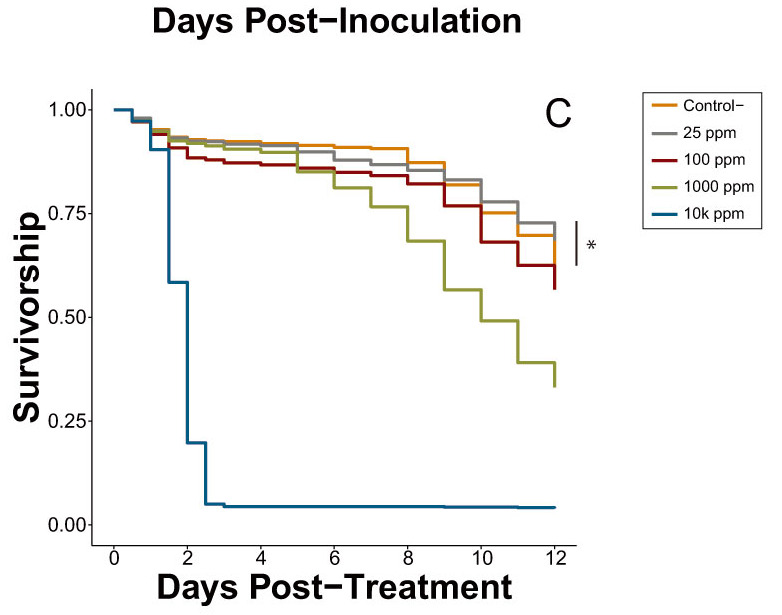
Average survival curves of all honey bee cages inoculated (+, dash lines) or uninoculated (−, solid lines) with IAPV and fed varying concentrations of caffeine during the caffeine investigation. (**A**) Bees were fed sucrose solution or 25 ppm caffeine, with caffeine+ bees experiencing significantly higher survival than control+ bees, *n* = 30 cages per treatment. All bees were either inoculated (**B**) or uninoculated (**C**) with IAPV and were fed sucrose or caffeine-sucrose solutions ranging from 25–10,000 (10k) ppm, *n* = 20 cages per treatment. Bees inoculated with IAPV (**B**) and fed 100 ppm caffeine showed increased survival compared to all other treatments while those fed unamended sucrose and 25 ppm caffeine showed no significant difference to one another. NS, not significant; * *p* < 0.05, ** *p* < 0.01, *** *p* < 0.001 (pairwise Cox proportional-hazards models, Benjamini-Hochberg correction).

The survival trends of the second set of caffeine trials (Experiment 5) remained consistent with those of the multi-phytochemical trial (Figure 1D). Combining the results of both trials does not significantly alter the magnitude of effect (Cox proportional-hazards models; HR = 0.86, *p* = 0.005; Figure 2A, Table S5), with the hazard ratios between inoculated caffeine and inoculated control treatments of both trials remaining within 95% confidence intervals of one another.

#### 3.2.2. Caffeine Dose-Response (Experiment 6)

In the caffeine dose-response trial with inoculated bees (Experiment 6A), 100 ppm caffeine significantly decreased mortality risk by 13.5% and 34.3% when compared to the inoculated control and 25 ppm caffeine, respectively (Cox proportional-hazards models; HR = 0.87, *p* = 0.002; HR = 0.66, *p* < 0.001; Table S6). The control and 25-ppm treatment were not significantly different from one another but there was a trend for bees in the 25-ppm treatment to experience an increased mortality risk (Cox proportional-hazards models; HR = 1.61, *p* = 0.083), a result directly in contrast to the results observed in previous caffeine trials (Figure 2). Both of the elevated dosages (1000 ppm and 10,000 ppm) significantly increased mortality risk when compared to all other treatments, with all cages with 10,000 ppm caffeine experiencing complete mortality by Day 3 (Table S6).

In the caffeine dose-response trial with uninoculated bees (Experiment 6B), the 25-ppm dose significantly decreased mortality risk by 21.6% when compared to the uninoculated control (Cox proportional-hazards models; HR = 0.78, *p* = 0.012; Figure 2C). This differentiation between the control and 25 ppm was not observed in the earlier caffeine trials (Experiments 4 and 5) between the same treatments tested under the same conditions. The remaining treatments each increased the mortality risk in the order of 10,000 ppm > 1000 ppm > 100 ppm > control, with 10,000 ppm dropping to near-complete mortality in all cages by Day 3 (Table S7).

#### 3.2.3. Caffeine Cages Viral Quantification

**Figure 3:**
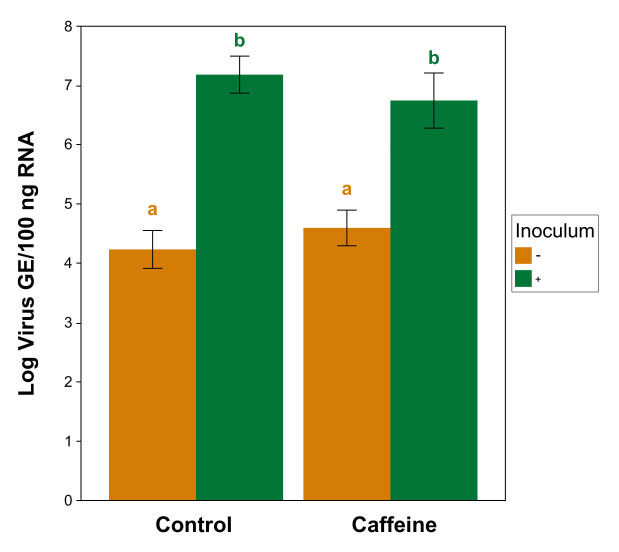
IAPV loads (log estimated genome equivalents per 100 ng total RNA) of bees sampled from the caffeine survival trials (Experiments 4 and 5) using RT-qPCR. Bars represent averages of pooled samples of inoculated (+) and uninoculated (−) treatments across both diet types (±1 standard error). Letters denote significant differences between groups, Kruskal-Wallis ANOVA, Wilcoxon rank-sum post-hoc test, Benjamini-Hochberg correction, *p* < 0.05. *n* = 8 for both inoculated treatments, *n* = 6 and 7 for caffeine- and control-treatments, respectively.

RT-qPCR confirmed the presence of IAPV infection in the inoculated treatments (Figure 3). The bees sampled from both inoculated treatments contained significantly higher viral loads than the uninoculated bees of either diet type (caffeine *p* = 0.019; control *p* = 0.004). However, no significant differences in viral loads were detected within the infection groups across the diet treatments (inoculated *p* = 0.645; uninoculated *p* = 0.641) (Kruskal-Wallis ANOVA, χ^2^ = 17.03, df = 3, *p* < 0.001; Wilcoxon rank-sum post-hoc; Benjamini-Hochberg corrections). Three cages were discarded from final analysis due to contamination, resulting in total *n* = 29 cages (caffeine − = 6, caffeine + = 8, control − = 7, control + = 8).

See Table 2 for a complete summary of all experiments and their associated figures and tables.

## 4. Discussion

Using a model virus survivorship bioassay, we provide evidence that honey bee mortality from virus infection can be ameliorated through feeding of natural-level concentrations of caffeine, though other phytochemicals did not show similar responses. The phytochemical screening process determined that neither of the two tested essential oils in their encapsulated forms is capable of ameliorating the effects of IAPV infection. These results were reflected in all tests for both phytochemicals, regardless of concentration (Figure 1A–C). Although carvacrol has been shown to have antibacterial properties [59,60], there has been little to no examination of carvacrol as an agent for pathogen treatment in honey bees. Our results, however, do not provide any evidence that encapsulated carvacrol affects virus infection in honey bees.

Thymol, on the other hand, has been shown to be an effective antipathogenic agent in *Apis* species, upregulates antimicrobial peptide expression in honey bees, and can suppress *Crithidia bombi* in bumble bees [22,23]. The acaricidal properties of thymol are well-documented and its use for *Varroa* mite control has increased in popularity with formulations such as Apiguard^®^ or ApiLife VAR^®^ [20,21]. The 160-ppm dose implemented in our thymol treatments trial (Experiment 3) falls within the range of thymol residue concentrations that can be found in hive wax after a standard two-week Apiguard^®^ treatment (147.7 ± 188.9 ppm) [20]. Despite this value being two orders of magnitude greater than the thymol concentration detected in honey in the same experiment (0.96 ± 0.61 ppm), a previous study has shown that honey bees can consume a similar thymol dose without negative effects. Indeed, Costa et al. [61] showed that bees fed 100-ppm thymol syrup not only survived significantly longer than control bees, they also had significantly reduced *Nosema ceranae* spore loads. Although it is possible the uninoculated 160-ppm thymol treatment could have yielded a similar longevity-boosting result had the thymol treatment experiment been extended past 11 days (a non-significant trend is visible in Figure 1C between 160 ppm- and control), neither the 160- nor the 1000-ppm concentration improved survival in the presence of IAPV infection (Figure 1C). Furthermore, thymol does not appear to prevent viral infection, as suggested by the two prophylactic treatments.

One possibility for future testing would be to reduce the amount of time during which both essential oils are consumed. The young bees observed to experience DWV-level reduction by Palmer-Young et al. [22] were fed thymol solution for only 24 h before being released into a hive—suggesting that the antiviral qualities of thymol may be effective only in the short term. Additionally, subsequent tests involving encapsulated essential oils should confirm whether or not the phytochemical was taken up by the honey bee itself. One major advantage of encapsulating essential oils is that the oligosaccharide coating masks the powerful odor of the phytochemical in its pure form, allowing for the administration of much higher doses that would otherwise be repellent to honey bees [52]. Although the coating surrounding the particles is easily broken down within a human digestive system [19], it is possible that the honey bee digestive tract might not be as adept at releasing encapsulated phytochemicals, and, thus, the bees may not be actually gaining any therapeutic benefits. Thymol is about 10 times more toxic to honey bees than carvacrol [62], yet the uninoculated bees in the 160- and 1000-ppm carvacrol treatments had roughly the same survival rate as the equivalent thymol treatments at the same time points (Figure 1A–C). However, the carvacrol and thymol treatments were never run concurrently with one another, so these similarities are based entirely on survival trends rather than an established lack of oligosaccharide release. In fact, the rapid decline of the uninoculated bees in the 1000 ppm thymol-fed cages relative to the bees in the uninoculated control cages in Experiment 3 suggests that thymol *was* being released and subsequently overdosing the bees (Figure 1C). Additionally, Ellis and Baxendale’s [62] assay was performed using phytochemical fumigants instead of direct ingestion, meaning that the magnitude of toxicity may not be directly translatable to the system we tested. Further investigations will be needed to validate the results obtained in our experiments on encapsulated carvacrol and thymol and IAPV infection.

The screening process also revealed that neither *p*-coumaric acid nor quercetin were capable of improving the survivorship of honey bees inoculated with IAPV (Figure 1D). Both of these phytochemicals possess multiple survivorship-boosting capabilities, including reducing *N. ceranae* spore load, upregulating detoxification genes, and increasing pesticide tolerance [25,26,27,29]. Furthermore, they both inhibit the replication and production of a large number of viruses, such as Canine Distemper Virus [30], human rhinovirus [31], herpes simplex virus [63], and Japanese encephalitis virus [64], although these tests have thus far been conducted only in mammalian systems. Our current results do not support the hypothesis that these phytochemicals help combat IAPV infection, but it is difficult to rule out whether or not they activate any of the various defense mechanisms honey bees utilize to defend against viral infection without subsequent molecular analyses.

Caffeine, however, was identified by the phytochemical screening process as the most promising potential agent for improving resilience against virus infection. The initial caffeine trials (Experiments 4 and 5) clearly showed it significantly increases the survival of IAPV-inoculated honey bees, thereby expanding the known repertoire of caffeine’s physiological effects in honey bee systems. Most notably, 25 ppm, a concentration that bees can and do encounter in naturally occurring floral resources [35,40], was sufficient to generate a beneficial effect. As documented previously [65], 1000 and 10,000 ppm caffeine concentrations were lethal, even in the absence of virus treatment (Figure 2B,C and Figure 3). In the inoculated dose-response (Experiment 6A), 100 ppm significantly improved survivorship compared to both the inoculated positive control and the 25-ppm treatment (Table S6). At 100 ppm, caffeine is still within its natural range found in *Citrus* flower nectar [34] and it is reasonable to expect an increased dose will amplify the antiviral effect, to a point. However, the results of the inoculated dose-response trial exhibited some key differences from those observed in the initial caffeine trials (Figure 2A). Despite identical bioassay methods, the 25-ppm caffeine treatment of Experiment 6A did not cause a significant difference in survival compared to the control. Additionally, the reduction in mortality risk observed in the 100-ppm treatment in Experiment 6A (13.7%) was nearly identical to that of the 25-ppm treatment in Experiment 5 (13.5%).

This change in concentration-based treatment effectiveness could potentially be attributed to a seasonally dependent nutritional dearth. Dolezal et al. [66] showed that hives situated in Midwestern agricultural landscapes (within which our bees were reared) frequently suffer from a lack of forage availability starting in early August, culminating in reduced colony weight and individual fat stores. While the bees tested in Experiments 4 and 5 were collected during late June and early July, the bees in Experiment 6 were gathered in mid- to late August when they may have already begun to experience a nutritional deficit. We posit that this forage dearth could have contributed to the change in caffeine-virus interaction observed in the caffeine dose-response trials, with the nutritionally stressed bees requiring a stronger dose of caffeine to achieve the same level of survivorship improvement seen earlier in the season. Therefore, while the trend remains the same across the season, the magnitude of effects shows variation. Future work will be necessary to explore the interaction between caffeine and other nutritional factors.

The bees in the control and 25-ppm treatments of the uninoculated caffeine dose-response assay (Experiment 6B) also yielded inconsistent results when compared to the same treatments in Experiments 4 and 5. Experiment 6B revealed that 25 ppm significantly decreased mortality risk by 21.6% compared to control (Figure 2C, Table S7), while earlier experiments showed no difference between these two treatments. This divergence in survival pattern may have been caused by background virus infection. The bees used in Experiment 6B were collected in late August, the same point at which *Varroa* populations typically tend to rise [67], increasing the chances of an elevated viral load not mediated by humans. Undetected viral infections could have depressed the survivorship of the supposedly uninfected control bees and the 25-ppm caffeine dose may have been just enough to ameliorate the viral effects without also poisoning the bees. Future experimentation examining caffeine dose-response of inoculated and uninoculated bees should include repeated tests across a broader temporal range to take into account any seasonal variation, as well as testing a narrower range of caffeine concentrations under 1000 ppm to obtain a clearer image of the stratification of dosage on antiviral effect.

Experiments 6A and 6B laid the groundwork for research in several promising directions. In all of the cage assays, we intentionally excluded the addition of an external protein source as a means of maximizing the chances of observing an effect. Pollen is a critical component of the bee diet, affecting many aspects of bee biology [68], including immunocompetence [12], pesticide tolerance [26], and response to virus infection. Dolezal et al. [13] showed that even the quality of pollen can influence mortality rates of bees infected with viruses. The exclusion of dietary pollen, therefore, prevents the accidental masking or augmentation of any potential effects incurred by phytochemical consumption, an observation that was shared by Palmer-Young et al. [22] when discussing the effects of phytochemicals on pathogen infection. However, following the confirmation of caffeine’s ameliorative qualities, subsequent studies should introduce a protein supplement to examine how it affects the caffeine-virus interaction.

Lastly, RT-qPCR analysis confirmed the presence of viral infection in the expected groups across Experiments 4 and 5, with the inoculated treatments of both diet types having significantly greater viral loads than their uninoculated counterparts (Figure 3). These data are important in addressing the question of whether caffeine influences the ability of honey bees to tolerate high virus titers or reduce their replication [69]. Infected bees from both dietary treatments did not exhibit differences in virus titer, suggesting that the threshold of virus titer necessary to cause mortality was not changed by caffeine (i.e., they do not tolerate higher levels). However, this pattern also suggests that, at least among the bees that died, caffeine did not enhance the ability of bees to reduce viral replication. Future experiments should include additional sampling at multiple timepoints on live and dead bees to fully understand this phenomenon.

The survivorship-enhancing effects we documented provide a useful comparison to caffeine tested in other insect systems, highlighting the range of coevolutionary relationships that bees have with the plants they use as nectar sources, relative to other insect nectarivores. Multiple studies have shown that caffeine possesses larvicidal properties when administered to *Aedes* mosquitoes [70,71], and Njoroge [72] demonstrated that concentrations similar to those that improved IAPV-inoculated bee survivorship (100, 200 ppm) significantly reduced the lifespan of *A. albopictus*. Eastep et al. [71] found that caffeine did not significantly alter the titers of the La Crosse virus (a mosquito-borne virus capable of causing encephalitis in humans) in *A. albopictus*, a result similar to that of IAPV measured in our experiments.

With its availability and already established familiarity, caffeine has potential as a relatively inexpensive and practical supplement to reduce virus-correlated mortality in managed colonies. However, it should be noted that, although caffeine clearly confers beneficial effects, it does not constitute a ‘medicine’ and therefore should not be counted on to completely clear viral infections. Instead, caffeine could serve as a useful instrument for furthering understanding of honey bee virus infections at the physiological and molecular level, while also acting as a potential dietary supplement. Larger scale experiments on whole colonies will be necessary to determine the ultimate effectiveness of such caffeine treatments, though field trials involving phytochemical supplements should take into consideration any national legal requirements regarding colony supplementation declaration. Ascertaining the full scope of caffeine’s antiviral qualities can help craft recommendations for reducing virus pressure in managed honey bee colonies, provide understanding of basic mechanisms by which bees reduce pathogen infection, and enrich the understanding of the coevolution between plant nectar sources and pollinators.

## 5. Conclusions

Through a series of screening experiments, we have shown that *p*-coumaric acid, quercetin, and encapsulated thymol and carvacrol do not have a significant effect on honey bee survivorship during IAPV infection, though the bioassay screens we performed do not account for all possible scenarios. Most notably, we have demonstrated that caffeine consumed at a naturally occurring level can significantly increase survival of honey bees when they are infected with IAPV. Furthermore, we expanded upon our initial caffeine results by establishing caffeine dose-response curves in honey bees to determine the scope of caffeine’s effect at different concentrations with or without viral infection. These dose-response curves show that 100 ppm caffeine has a similar effect to that of 25 ppm in IAPV-inoculated bees, but only 25 ppm improves survivorship relative to controls fed unamended sucrose in the absence of IAPV inoculation. We recommend additional testing to fully determine the usefulness of caffeine as a honey bee antiviral agent and to improve comprehension of virus–phytochemical interactions in general.

## Figures and Tables

**Table 1:** Summary of thymol acute rescue experiment details. IAPV column denotes presence (+) or absence (−) of a 1% Israeli acute paralysis virus (IAPV) inoculum.

Treatment	Thymol Concentration	IAPV (1%)	
Control–standard	0 ppm	−	
*Ad libitum*	160 ppm	−	+
Acute Response	1000 ppm	−	+
Prophylactic–Control	0 ppm; 0 ppm	+	
Prophylactic–Inoculated	160 ppm; 0 ppm	+	

**Table 2:** Reference table describing all experiments and their associated figures and hazard ratios.

Experiment	Description	Figure	Table(s)
Exp. 1	Carvacrol moderate dose treatments	Figure 1A	Table S1
Exp. 2	Carvacrol acute dose treatments (1000–2000 ppm)	Figure 1B	Table S2
Exp. 3	Thymol treatments (acute dose, prophylactic, 160 ppm)	Figure 1C	Table 1 and Table S3
Exp. 4	Multi-phytochemical trial (quercetin, PCA, caffeine)	Figure 1D	Table S4
Exp. 5	All 25-ppm caffeine treatments	Figure 2A	Table S5
Exp. 6A	Inoculated caffeine dose-response trial	Figure 2B	Table S6
Exp. 6B	Uninoculated caffeine dose-response trial	Figure 2C	Table S7
Virus Quant.	Viral titers of Experiments 6 and 7 via RT-qPCR	Figure 3	-

## Supplementary Materials

The following are available online at https://www.mdpi.com/2075-4450/11/10/698/s1, Table S1: Hazard ratios of pairwise Cox proportional-hazards model comparisons between treatments in the carvacrol treatment trial (Exp. 1); Table S2: Hazard ratios (HR) of pairwise Cox proportional-hazards model comparisons between treatments in the carvacrol acute rescue trial (Exp. 2); Table S3: Hazard ratios (HR) of pairwise Cox proportional-hazards model comparisons between treatments in the thymol trial (Exp. 3); Table S4: Hazard ratios (HR) of pairwise Cox proportional-hazards model comparisons between treatments in the multi-phytochemical trial (Exp. 4); Table S5: Hazard ratios (HR) of pairwise Cox proportional-hazards model comparisons between treatments in the caffeine only trials (Exp. 5 & part of Exp. 4); Table S6: Hazard ratios (HR) of pairwise Cox proportional-hazards model comparisons between treatments in the inoculated caffeine dose-response trial (Exp. 6A); Table S7: Hazard ratios (HR) of pairwise Cox proportional-hazards model comparisons between treatments in the uninoculated caffeine dose-response trial (Exp. 6B).
