# Supplementary material for: Ameliorative Effects of Phytochemical Ingestion on Viral Infection in Honey Bees
Source: Insects. 2020 Oct 13;11(10):698. doi: 10.3390/insects11100698 (PMC7602108; doi:10.3390/insects11100698)
Supplement: Supplementary file 1 [file insects-11-00698-s001.pdf]

Supplemental Tables:

Table 1. Hazard ratios (HR) of pairwise Cox proportional-hazards model comparisons between treatments in the carvacrol treatment trial (Experiment 1). Blue and red HRs indicate significant increased and decreased risk of death, respectively (***p < 0.001; Benjamini-Hochberg correction). Comparisons are read from left-row to top-column; e.g. Row 2 treatment increases or decreases risk of death by a factor of *x* when compared to Column 3. + (Israeli acute paralysis virus (IAPV) inoculated), - (uninoculated); Control = sucrose only, CV = 160 ppm carvacrol; N/A = not applicable. *n* = 7 cages for all treatments.

Treatment	Control-	CV-	CV+
Control-	N/A	N/A	N/A
CV-	0.606	N/A	N/A
CV+	4.199***	28.465***	N/A
Control+	2.580***	5.274***	1.005

Table 2. Hazard ratios (HR) of pairwise Cox proportional-hazards model comparisons between treatments in the carvacrol acute rescue trial (Experiment 2). Blue and red HRs indicate significant increased and decreased risk of death, respectively (**p < 0.01, ***p < 0.001; Benjamini-Hochberg correction). Comparisons are read from left-row to top-column; e.g. Row 2 treatment increases or decreases risk of death by a factor of *x* when compared to Column 3. + (IAPV inoculated), - (uninoculated); Control = sucrose only. *n* = 7 cages for all treatments.

Treatment	Control	1000 ppm-	1000 ppm+	2000 ppm-
Control	N/A	N/A	N/A	N/A
1000 ppm-	1.677	N/A	N/A	N/A
1000 ppm+	7.559***	34.276***	N/A	N/A
2000 ppm-	2.029***	2.215***	0.111***	N/A
2000 ppm+	2.560***	2.955***	0.897**	6.372***

Table 3. Hazard ratios (HR) of pairwise Cox proportional-hazards model comparisons between treatments in the thymol trial (Experiment 3). Blue and red HRs indicate significant increased and decreased risk of death, respectively (**p < 0.01, ***p < 0.001; Benjamini-Hochberg correction). Comparisons are read from left-row to top-column; e.g. Row 2 treatment increases or decreases risk of death by a factor of *x* when compared to Column 3. + (IAPV inoculated), - (uninoculated); Control = sucrose only, ProCon = prophylactic fed sucrose only, ProTY = prophylactic fed thymol then sucrose; *n* = 7 cages for all other treatments.

Treatment	Control	160 ppm-	160 ppm+	1000 ppm-	1000 ppm+	ProCon+
Control	N/A	N/A	N/A	N/A	N/A	N/A
160 ppm-	0.658	N/A	N/A	N/A	N/A	N/A
160 ppm+	2.923***	12.697***	N/A	N/A	N/A	N/A
1000 ppm-	2.078***	3.770***	0.595***	N/A	N/A	N/A
1000 ppm+	1.951***	2.810***	1.148**	2.690***	N/A	N/A
ProCon+	1.433***	1.773***	0.790***	0.812***	0.296***	N/A
ProTY+	1.372***	1.615***	0.842***	0.910**	0.560***	1.157

Table 4. Hazard ratios (HR) of pairwise Cox proportional-hazards model comparisons between treatments in the multi-phytochemical trial (Experiment 4). Blue and red HRs indicate significant increased and decreased risk of death, respectively (*p < 0.05, **p < 0.01, ***p < 0.001; Benjamini-Hochberg correction). Comparisons are read from left-row to top-column; e.g. Row 2 treatment increases or decreases risk of death by a factor of *x* when compared to Column 3. + (IAPV inoculated), - (uninoculated); Control = sucrose only, Caff = caffeine, PCA = *p*-coumaric acid, Quer = quercetin. *n* = 9 cages for Caff+; *n* = 10 cages for all other treatments.

**Treatment**	**Control-**	**Control+**	**Caff-**	**Caff+**	**PCA-**	**PCA+**	**Quer-**
Control-	N/A	N/A	N/A	N/A	N/A	N/A	N/A
Control+	6.416***	N/A	N/A	N/A	N/A	N/A	N/A
Caff-	1.101	0.185***	N/A	N/A	N/A	N/A	N/A
Caff+	1.561***	0.781**	3.254***	N/A	N/A	N/A	N/A
PCA-	1.034	0.547***	0.920	0.228***	N/A	N/A	N/A
PCA+	1.440***	0.948	1.743***	1.245***	7.223***	N/A	N/A
Quer-	0.870	0.585***	0.755*	0.460***	0.637	0.058***	N/A
Quer+	1.297***	0.971	1.390***	1.112***	1.882***	0.978	15.884***

Table 5. Hazard ratios (HR) of pairwise Cox proportional-hazards model comparisons between treatments in the caffeine only trials (Experiment 5 & part of Expermient 4). Note this table contains the control and caffeine data from Table B. Blue and red HRs indicate significant increased and decreased risk of death, respectively (**p < 0.01, ***p < 0.001; Benjamini-Hochberg correction). Comparisons are read from left-row to top-column; e.g. Row 2 treatment increases or decreases risk of death by a factor of *x* when compared to Column 3. + (IAPV inoculated), - (uninoculated); Control = sucrose only, Caff = 25 ppm caffeine. *n* = 29 cages for Caff+, 30 cages for all other treatments.

**Treatment**	**Control-**	**Control+**	**Caff-**
**Control-**	N/A	N/A	N/A
**Control+**	6.423***	N/A	N/A
**Caff-**	1.117	0.200***	N/A
**Caff+**	1.690***	0.863**	3.554***

Table 6. Hazard ratios (HR) of pairwise Cox proportional-hazards model comparisons between treatments in the inoculated caffeine dose-response trial (Experiment 6A). Blue and red HRs indicate significant increased and decreased risk of death, respectively (**p < 0.01, ***p < 0.001; Benjamini-Hochberg correction). Comparisons are read from left-row to top-column; e.g. Row 2 treatment increases or decreases risk of death by a factor of *x* when compared to Column 3. Control = sucrose only. *n* = 20 cages per treatment.

**Treatment**	**Control**	**25 ppm**	**100 ppm**	**1000 ppm**
Control	N/A	N/A	N/A	N/A
25 ppm	1.612	N/A	N/A	N/A
100 ppm	0.865**	0.657***	N/A	N/A
1000 ppm	1.133***	1.111**	1.892***	N/A
10k ppm	1.639***	1.771***	3.175***	4.722***

Table 7. Hazard ratios (HR) of pairwise Cox proportional-hazards model comparisons between treatments in the uninoculated caffeine dose-response trial (Experiment 6B). Blue and red HRs indicate significant increased and decreased risk of death, respectively (*p < 0.05, **p < 0.01, ***p < 0.001; Benjamini-Hochberg correction). Comparisons are read from left-row to top-column; e.g. Row 2 treatment increases or decreases risk of death by a factor of *x* when compared to Column 3. Control = sucrose only. *n* = 20 cages per treatment.

**Treatment**	**Control**	**25 ppm**	**100 ppm**	**1000 ppm**
Control	N/A	N/A	N/A	N/A
25 ppm	0.784*	N/A	N/A	N/A
100 ppm	1.105**	1.516***	N/A	N/A
1000 ppm	1.323***	1.686***	1.894***	N/A
10k ppm	1.947***	2.494***	3.346***	8.125***
